# The Oligo Fucoidan Inhibits Platelet-Derived Growth Factor-Stimulated Proliferation of Airway Smooth Muscle Cells

**DOI:** 10.3390/md14010015

**Published:** 2016-01-09

**Authors:** Chao-Huei Yang, Chiung-Fang Tsao, Wang-Sheng Ko, Ya-Ling Chiou

**Affiliations:** 1 Department of Internal Medicine, Kuang-Tien General Hospital, No. 117, Shatian Road Shalu District, Taichung City 433, Taiwan; yan5104@ms23.hinet.net; 2 Department of Biotechnology, Hungkuang University, 34 Chung-Chie Rd, Sha Lu, Taichung 443, Taiwan; 717230@gmail.com; 3 Institute of BioMedical Nutrition, Hungkuang University, 34 Chung-Chie Rd, Sha Lu, Taichung 443, Taiwan; 4 Department of Nursing, Hungkuang University, 34 Chung-Chie Rd, Sha Lu, Taichung 443, Taiwan

**Keywords:** asthma, airway smooth muscle cells, oligo-fucoidan

## Abstract

In the pathogenesis of asthma, the proliferation of airway smooth muscle cells (ASMCs) is a key factor in airway remodeling and causes airway narrowing. In addition, ASMCs are also the effector cells of airway inflammation. Fucoidan extracted from marine brown algae polysaccharides has antiviral, antioxidant, antimicrobial, anticlotting, and anticancer properties; however, its effectiveness for asthma has not been elucidated thus far. Platelet-derived growth factor (PDGF)-treated primary ASMCs were cultured with or without oligo-fucoidan (100, 500, or 1000 µg/mL) to evaluate its effects on cell proliferation, cell cycle, apoptosis, and Akt, ERK1/2 signaling pathway. We found that PDGF (40 ng/mL) increased the proliferation of ASMCs by 2.5-fold after 48 h (*p* < 0.05). Oligo-fucoidan reduced the proliferation of PDGF-stimulated ASMCs by 75%–99% after 48 h (*p* < 0.05) and induced G_1_/G_0_ cell cycle arrest, but did not induce apoptosis. Further, oligo-fucoidan supplementation reduced PDGF-stimulated extracellular signal-regulated kinase (ERK1/2), Akt, and nuclear factor (NF)-κB phosphorylation. Taken together, oligo-fucoidan supplementation might reduce proliferation of PDGF-treated ASMCs through the suppression of ERK1/2 and Akt phosphorylation and NF-κB activation. The results provide basis for future animal experiments and human trials.

## 1. Introduction

In 1974, the morbidity rate of childhood asthma in Taiwan was approximately 3%, which increased to approximately 20.74% by 2009, indicating a 7-fold increase in 35 years [1] This shows that asthma has become a common and serious issue Taiwan. In the pathogenesis of asthma, alteration in the structure of the airways is commonly observed resulting in airway smooth muscle cell hypertrophy, hyperplasia, and airway wall thickening. Recent studies have demonstrated that airway smooth muscle cells (ASMCs) are not just involved in airway narrowing. Stimulated ASMCs release interleukin (IL)-1, IL-5, IL-6, IL-8, IL-11, granulocyte monocyte-colony stimulating factor (GM-CSF), leukemia inhibitory factor (LIF), and monocyte chemoattractant protein (MCP)-1, 2, 3. The stimulated ASMCs also express cell adhesion molecules (CAMs), intercellular CAM (ICAM), and vascular CAM (VCAM) to activate mast cells, eosinophils, lymphocytes, and neutrophil causing airway inflammation [2]; thus, the majority of asthma patients require permanent therapy with inhaled corticosteroids (ICS) or ICS + long-acting beta2-agonists (LABA) to ameliorate airway inflammation [3]. Therefore, reducing the proliferation of ASMCs is one approach of reducing airway inflammation. In asthma pathogenesis, the injured epithelial tissue releases several factors, including endothelin-1, epidermal growth factor, insulin-like growth factor, and platelet-derived growth factor (PDGF) to induce the proliferation of ASMCs [4,5,6]. PDGF is a major factor in proliferation of ASMCs [7,8,9]. The PDGF family consists of five different isoforms, including PDGF-AA, PDGF-AB, PDGF-BB, PDGF-CC, and PDGF-DD [10]. Both *in vivo* and *in vitro* studies showed that PDGF-BB induced the proliferation and migration of human ASMCs [11,12,13]. Therefore, the increased level of PDGF-BB in the airway of asthmatic patients was associated with changes in airway structure and function. PDGF can induce proliferation of ASMCs in a dose-dependent manner by activating various signaling pathways, including the mitogen activated protein kinase (MAPK), phosphoinositide-3-kinase (PI3K), and nuclear factor (NF)-κB pathways [14,15,16]. The proliferation of ASMCs is believed to be important in causing airway hyper-responsiveness (AHR), a prominent feature of airway remodeling [9].

Fucoidan is a seaweed polysaccharide derived from brown seaweed extract. It is structurally similar to heparin and consists of an alpha-1,3-backbone or a repeat units of disaccharides containing alpha-1,3-linked fucose and an alpha-1,4-linked fucose, with branching at the C2 position [17,18]. The fucoidan polysaccharide is reported to have antiviral, antioxidant, antimicrobial, anticoagulant, anticancer/antitumor, antiproliferative, and anti-inflammatory properties [18,19]. The use of fucoidan from various seaweed extracts for atopic allergic reactions was found to enhance the natural immune response, alter Th1/Th2 balance, inhibit immunoglobulin E (IgE) production, and suppress mast cell degranulation. Because fucoidan has these immunomodulatory effects, it has the potential to prevent allergic diseases [20,21]. However, the effects of oligo-fucoidan on allergic disease have not been elucidated thus far.

The proliferation of ASMCs is a key factor in causing AHR. In ASMCs, the regulation of cell proliferation signals closely influences pathological processes. Therefore, we aimed to investigate the effects of oligo-fucoidan on the proliferation of PDGF-treated ASMCs. We used PDGF to induce the proliferation of ASMCs and found that treatment with oligo-fucoidan (100, 500, or 1000 µg/mL) inhibited the proliferation of PDGF-treated ASMCs. Oligo-fucoidan inhibited ERK1/2, Akt, and NF-κB phosphorylation in PDGF-treated ASMCs. Therefore, oligo-fucoidan may decrease the proliferation of PDGF-treated ASMCs by suppressing the ERK1/2, Akt, and NF-κB pathways.

## 2. Results

### 2.1. Oligo-Fucoidan Inhibited PDGF-Stimulated Proliferation of ASMCs

**Figure 1:**
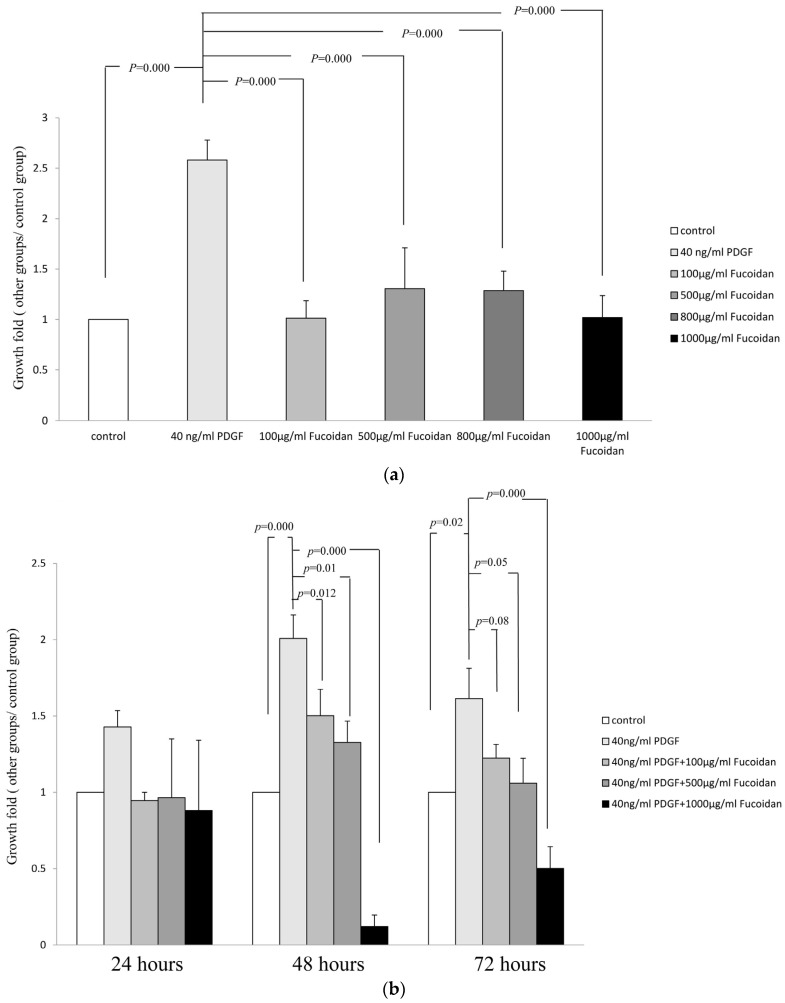

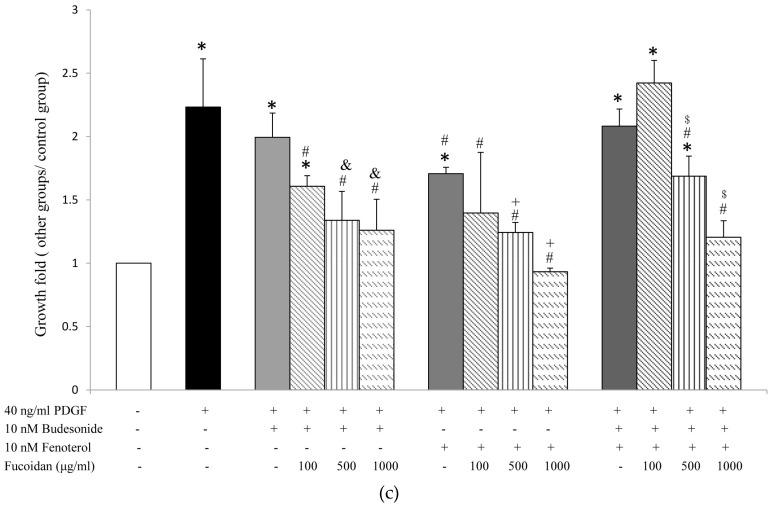
The oligo Fucoidan inhibited platelet-derived growth factor (PDGF)-stimulated proliferation of airway smooth muscle cells (ASMCs). ASMCs were serum deprivation (0.2% FBS-containing medium) for 48 h, growth-arrested ASMCs were treated with 40-ng/mL rhPDGF-BB or oligo fucoidan (100, 500, or 1000 µg/mL). After incubation for 48 h, the rate of proliferation of cells was assayed by XTT. (**a**) Growth-arrested cells were treated with 40-ng/mL rhPDGF-BB and oligo fucoidan (100, 500, or 1000 µg/mL) for 24 h, 48 h, or 72 h, the rate of proliferation of cells was assayed by XTT; (**b**) Growth-arrested cells were treated with 40-ng/mL rhPDGF-BB, 40-ng/mL rhPDGF-BB + 10 nM budesonide, 40-ng/mL rhPDGF-BB + 10 nM fenoterol and 40-ng/mL rhPDGF-BB + combination (10 nM budesonide + 10 nM fenoterol) withot or with different oligo fucoidan (100, 500, or 1000 µg/mL) concentration for 48 h, the rate of proliferation of cells was assayed by XTT.* compare with control group (*p* < 0.05); # compare with PDGF group (*p* < 0.05); & compare with PDGF + budesonide group (*p* < 0.05); + compare with PDGF + fenoterol group (*p* < 0.05), $ compare with PDGF + budesonide + fenoterol group (*p* < 0.05); (**c**) Results are from three individual experiments. One-way ANOVA and post hoc Tukey’s test have been used. *p* < 0.05 indicated significant differences.

To evaluate the effect of oligo-fucoidan on PDGF-stimulated proliferation of ASMCs, growth-arrested ASMCs were treated with 40-ng/mL rhPDGF-BB or oligo fucoidan (100, 500, or 1000 µg/mL). After incubation for 48 h, PDGF at 40-ng/mL stimulated marked proliferation of ASMCs. No significant differences in the proliferation rate of oligo-fucoidan alone treated ASMCs compared to the control group. Treatment with oligo-fucoidan alone did not affect the growth of cells cultured in 0.2% FBS-containing medium. (Figure 1a). Growth-arrested cells were treated with 40-ng/mL PDGF and oligo-fucoidan (100, 500, or 1000 µg/mL) for 24 h, 48 h, or 72 h. The proliferation fold of ASMCs decreased in 40 ng/mL PDGF and oligo-fucoidan groups significantly (Figure 1b). These results indicated that oligo-fucoidan inhibited the proliferation of PDGF-stimulated ASMCs. To evaluate the effect of oligo-fucoidan cpmpare with budesonide and fenoterol on PDGF-stimulated proliferation of ASMCs, Growth-arrested cells were treated with 40-ng/mL PDGF, 10 nM budesonide, 10 nM fenoterol and oligo-fucoidan (100, 500, or 1000 µg/mL) for 24 h and 48 h. The proliferation fold of PDGF-stumlated ASMCs did not decrease in budesonide and combination groups (10 nM budesonide + 10 nM fenoterol), except fenoterol group (*p* < 0.05) (Figure 1c). However, the combinatic effect of oligo-fucoidan (500 or 1000 µg/mL) reduced the proliferation fold of PDGF-stumlated ASMCs in budesonide, fenoterol and combination groups significantly (*p* < 0.05). These results indicated that oligo-fucoidan significantly improve the effects of budesonide and fenoterol in the proliferation of PDGF-stimulated ASMCs.

### 2.2. Oligo-Fucoidan Inhibited Cell Cycle Progression, but Did Not Induce Apoptosis in PGDF-Stimulated ASMCs

**Figure 2:**
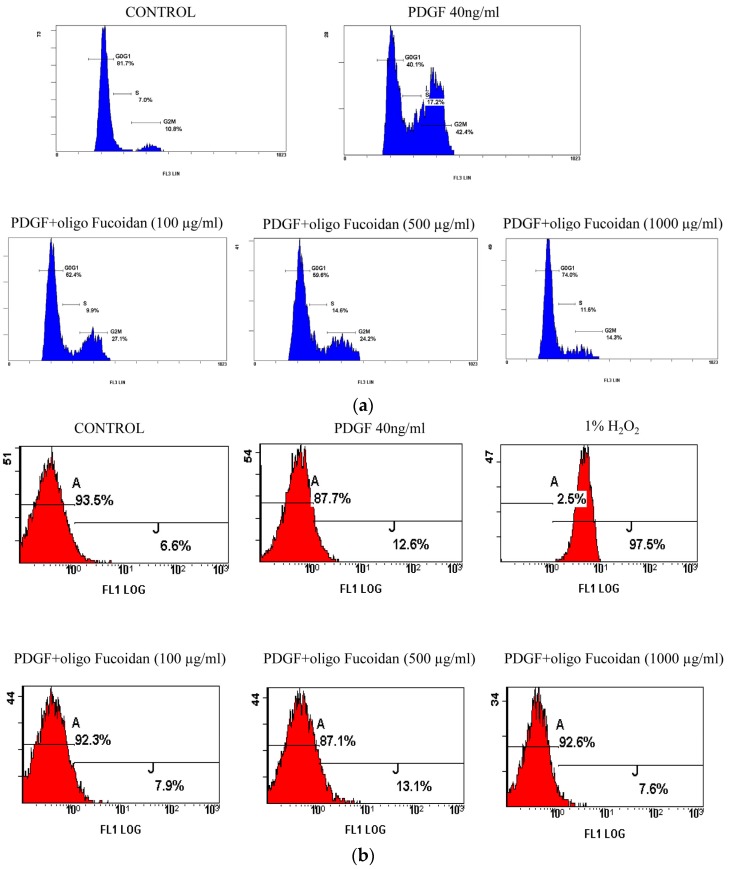
Oligo fucoidan inhibited the cell cycle progression, but did not induce apoptosis, in PGDF-stimulated ASMCSs. The growth-arrested ASMCs were stimulated with 40-ng/mL PDGF and oligo fucoidan (100, 500 and 1000 µg/mL) for 24 h. The cells were obtained to stain propidium iodide and assay cell cycle profiles by flow cytometry. (**a**) The growth-arrested ASMCs were stimulated with 40-ng/mL PDGF and oligo fucoidan (100, 500 and 1000 µg/mL) for 48 h. The growth-arrested ASMCs were stimulated with 40-ng/mL PDGF for 48 h and 1% H_2_O_2_ for 10 min as positive control group. The cells were obtained to stain Annexin V/propidium iodide and assay apoptosis ratio by flow cytometry; (**b**) One representative example of three individual experiments. One-way ANOVA and post hoc Tukey’s test have been used. *p* < 0.05 indicated significant differences.

We elucidated the effects of oligo-fucoidan on cell cycle progression by using growth-arrested cells that were stimulated with PDGF in the presence or absence of oligo-fucoidan (100 or 500 µg/mL) for 24 h and obtained cell cycle profiles by flow cytometric analysis. When growth-arrested ASMCs were stimulated with PDGF for 24 h, only 40.1% of the cells were in the G_1_/G_0_ phase (G_1_/G_0_) phase in control group (81.7%) (*p* < 0.05). Oligo-fucoidan significantly inhibited the PDGF-stimulated G1-to-S progression, resulting in 62.4%–74% of cells in the G_1_/G_0_ phase (Figure 2a). The percentage of apoptotic cells was determined by Annexin V-FITC apoptosis detection kit (Strong Biotech Corporation, Taipei, Taiwan). When growth-arrested ASMCs were stimulated with PDGF for 48 h, Oligo-fucoidan did not increase apoptosis compare with control group (Figure 2b). The results indicated that oligo-fucoidan did not induce an apoptotic response. These results confirmed that the antiproliferative effects of oligo-fucoidan could not be attributed to its cytotoxicity. Oligo-fucoidan inhibited the proliferation of cells by inducing cell cycle arrest and not by inducing apoptosis or necrosis.

### 2.3. Oligo-Fucoidan Effectively Blocked the PDGF-Induced Akt and ERK1/2 Phosphorylation

**Figure 3:**
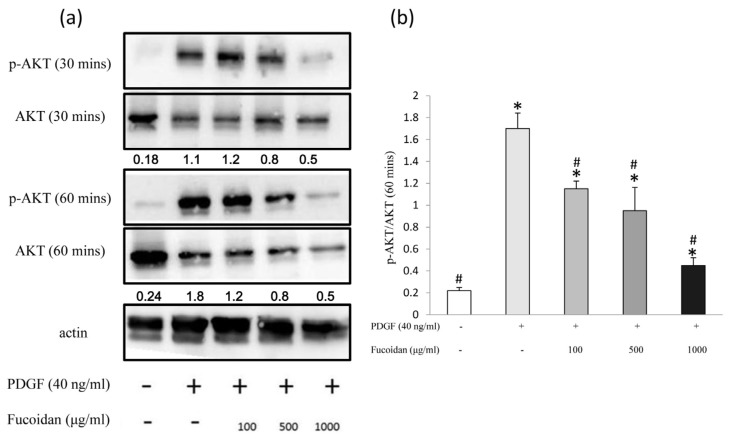
Oligo fucoidan decreased the PDGF-induced phosphorylation of Akt in ASMCs. Growth-arrested cells were treated with 40-ng/mL rhPDGF-BB or oligo fucoidan (100, 500, or 1000 µg/mL) for 30 and 60 min. Western blot was performed with phosphorylated Akt and Akt antibodies. Normalized signals (Akt phosphorylation/Akt) also were performance. (**a**) Normalized signals from three independent experiments are presented in the bar charts. * Significantly different from the value obtained with control group, # Significantly different from the value obtained with PDGF group, *p* < 0.05; (**b**) One-way ANOVA and post hoc Tukey’s test have been used. *p* < 0.05 indicated significant differences.

**Figure 4:**
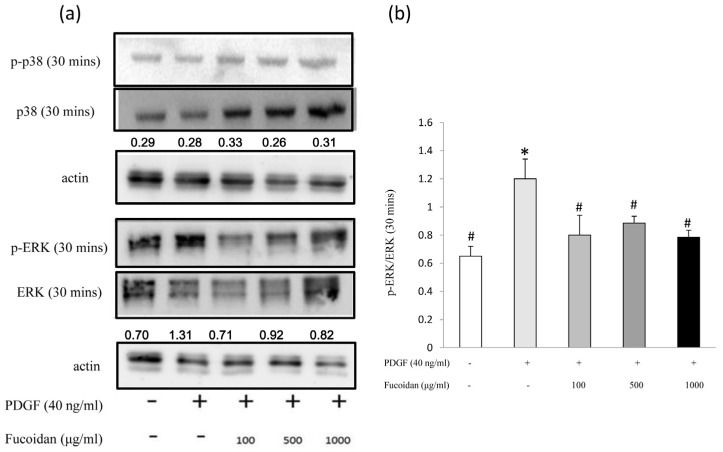
Oligo fucoidan effectively blocked the PDGF-induced ERK1/2 phosphorylation in ASMCs. Growth-arrested cells were treated with 40-ng/mL rhPDGF-BB or oligo fucoidan (100, 500, or 1000 µg/mL) for 30 min. Western blot was performed with phosphorylated p38, p38, phosphorylated ERK1/2 and ERK1/2 antibodies. Normalized signals (p38 phosphorylation/p38 and ERK1/2 phosphorylation/ERK1/2) also were performance. One representative example of three individual experiments. (**a**) Normalized signals from three independent experiments are presented in the bar charts. * Significantly different from the value obtained with control group; # Significantly different from the value obtained with PDGF group, *p* < 0.05; (**b**) One-way ANOVA and post hoc Tukey’s test have been used. *p* < 0.05 indicated significant differences.

We investigated the mechanisms by which oligo-fucoidan suppressed cell proliferation. PDGF stimulates the proliferation of ASMC through PI3 K signaling pathway, which can activate Akt protein. Activated-Akt in turn activates the downstream signaling proteins (p70S6 K, NF-κB, and ERK), increasing cyclin D1 level and suppressing the transcription of p27^Kip1^ to enable cell cycle progressionentry [22]. In this study, fucoidan inhibited Akt phosphorylation (Figure 3). Furthermore, PDGF not only induces Akt phosphorylation (Ser473) but also induces ERK1/2 phosphorylation to increase cell proliferation [22,23,24]. Our results also showed that oligo-fucoidan inhibited ERK1/2 phosphorylation, but not p38 phosphorylation (Figure 4).

### 2.4. Oligo-Fucoidan Effectively Blocked the PDGF-Induced NF-κB Phosphorylation

**Figure 5:**
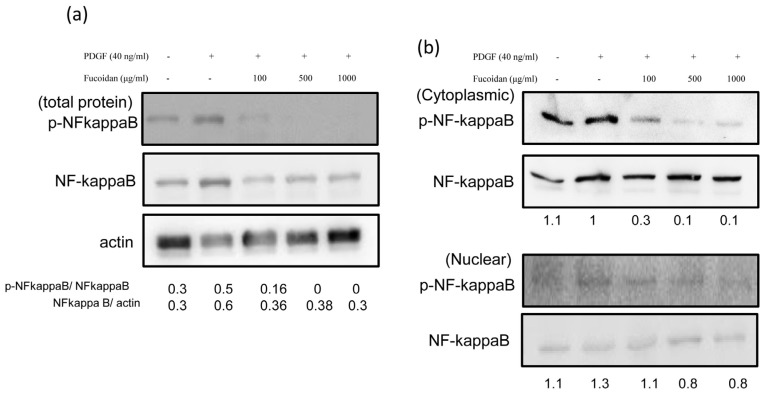
Oligo fucoidan effectively blocked the PDGF-induced NF-κB phosphorylation. Growth-arrested cells were treated with 40-ng/mL rhPDGF-BB or oligo fucoidan (100, 500, or 1000 µg/mL) for 30 min. Western blot was performed with phosphorylated NF-κB, NF-κB and actin antibodies in in total protein fractions (**a**) and in both cytoplasm and nuclear protein fractions (**b**). Normalized signals (NF-κB phosphorylation/NF-κB) also were performance. One representative example of three individual experiments.

We investigated whether oligo-fucoidan treatment induced changes in NF-κB activation. Growth-arrested cells were stimulated with PDGF in the presence or absence of oligo-fucoidan for 30 min. The densities of NF-κB in total protein fractions in various groups were compared with the level of phosphorylated NF-κB. Oligo-fucoidan markedly inhibited the level of phosphorylated-NF-κB (Figure 5a). NF-κB translocation was determined in both cytoplasm and nuclear protein fractions. PDGF increased the nuclear translocation of NF-κB after treatment for 30 min (Figure 5b).

## 3. Discussion

Asthma is subdivided into three phenotypes: (I) trigger-induced asthma, including (1) allergic; (2) non-allergic; (3) aspirin-exacerbated respiratory disease; (4) infection-; and (5) exercise-induced asthma. (II) Clinical presentation of asthma, including (1) pre-asthma wheezing in infants; (2) exacerbation-prone asthma; (3) associated with apparent irreversible airflow limitation. (III) Inflammatory markers of asthma [25]. In the pathogenesis of the three asthmatic phenotypes, structural changes in the airways are commonly observed resulting in airway smooth muscle cell hypertrophy, hyperplasia, and airway wall thickening. This study was the first report on the effects of fucoidan in airway smooth muscle cell hyperplasia. Fucoidan-based therapy could be beneficial for asthma per these data.

In the pathogenesis of asthma, the proliferation of ASMCs is a key factor in airway remodeling. Moreover, ASMCs are a source of chemokines and cytokines because they increase the recruitment and activation of inflammatory cells involved in airway inflammation [26]. A study on cultured ASMCs showed the phenotypic plasticity of ASMCs, which can transiently switch between a contractile (functional) and synthetic phenotype (proliferative) [27]. Normal ASMCs have the ability to contract; however, after exposure to stimulators (such as serum and growth factors) during growth, particularly in late passage, smooth muscle cells become increasingly irreversibly synthetic and 20% to 25% of the cells become hypercontractile. This phase of the cells is irreversible. We can speculate that “myofibroblasts” are phenotypically derived from ASMCs, perhaps in the guise of the synthetic phenotype [28].

Stimulated ASMCs caused airway inflammation [2]; thus, the majority of asthma patients require permanent therapy with inhaled corticosteroids (ICS) or ICS + long-acting beta2-agonists (LABA) to ameliorate airway inflammation [3]. In reducing proliferation of airway smooth muscle cells, some studies indicated ICS or ICS + LABA reduced mass of airway smooth muscle cells [29], we also determined the effects of Fucoidan combined with budesonide (inhaled corticosteroids), fenoterol (LABA) or budesonide + fenoterol in growth factor (PDGF)-stimulated airway smooth muscle cells. We found the Fucoidan combined with 10 nM budesonide (this dose has been over the recommended dose of inhaled budesonide), 10 nM fenoterol or 10 nM budesonide + 10 nM fenoterol more decreased PDGF-induced cell proliferation than budesonide, fenoterol or budesonide + fenoterol groups (Figure 1c). Fucoidan might be as an alternative or add-on therapy in proliferation of ASMCs. Fucoidan also has an anti-inflammation effect [18,19]. Therefore, as one of the future research directions, we will stimulate smooth muscle cells to produce inflammation, then discuss fucoidan and ICS and/or LABA effects. Fucoidan might be as an alternative or add-on therapy in airway inflammation.

There are several *in vivo* and *in vitro* plant extract studies directed at reducing inflammatory response in asthma [18,19,20]; however, there are limited studies focusing on the inhibition of ASMC proliferation. In this study, we evaluated the effects of oligo-fucoidan on primary cultured ASMCs. We showed that oligo-fucoidan inhibited the hyperplasia of ASMCs (Figure 1), but did not induce apoptosis, by stimulating PDGF (Figure 2).

Marine polysaccharides (fucoidan) have numerous pharmaceutical properties such as antioxidant, anti-inflammatory, antiallergic, antitumor, antiobesity, antidiabetes, anticoagulant, antiviral, immunomodulatory, cardioprotective, antihepatopathy, antiuropathy, and antirenalpathy activities [30]. Oligo-fucoidan has been characterized as a heparin-like molecule [8,9]. Previous studies have determined the pharmacological effects of heparin on the proliferation of ASMCs. The antiproliferative effects of heparin were reproduced by at least 3-kDa heparin and O-desulfated heparin (Ode; non-anticoagulant) [31,32]. However, the mechanism underlying the antiproliferative effects of heparin on ASMCs is not completely understood. In the present study, we demonstrated that oligo-fucoidan could significantly decrease PDGF-stimulated cell proliferation. Oligo-fucoidan suppressed Akt and ERK1/2 activation in PDGF-stimulated ASMCs (Figure 3 and Figure 4). These suppressions were correlated with the downregulation of the NF-κB signaling pathway (Figure 5). We found that oligo-fucoidan might decrease Akt and ERK1/2 phosphorylation to suppress NF-κB in primary cultured ASMCs by stimulating PDGF, thereby decreasing cell proliferation.

This study proposed that oligo-fucoidan inhibited the proliferation of ASMCs and achieved the goal of attenuating AHR. We found that PDGF induced the proliferation of ASMCs and oligo-fucoidan inhibited PDGF-stimulated cell proliferation in a dose-dependent manner by blocking the Akt, ERK1/2, and NF-κB signaling pathways. These effects may contribute to the effectiveness of oligo-fucoidan in attenuating AHR and improving airway remodeling. These results demonstrate that oligo-fucoidan serves as a potential complementary therapy for asthma.

Recently, fucoidan has been found to be a modulator of allergic responses by enhancing the natural immune response, altering Th1/Th2 balance, inhibiting IgE production, and suppressing mast cell degranulation. Our research specifically focused on the immunomodulatory effect of marine polysaccharides and emphasized on their potential application as pharmaceuticals and nutraceuticals for the prevention of allergic disorders. The immunomodulatory effect of fucoidan in allergic response should be further investigated. In other studies, fucoidan inhibited tumor necrosis factor (TNF)-alpha-stimulated NF-κB activity. NF-κB has been previously implicated in allergic dermatitis [33]. Our study demonstrates that oligo-fucoidan is a potential therapeutic supplement for asthma therapy and acts as a model for future protocol design in animal and preclinical studies. In the future, we plan to determine whether fucoidan can reduce experimental asthma injury by studying its effects on inflammation in ASMCs and in animal models of asthma.

## 4. Materials and Methods

### 4.1. Culture of ASMCs

Adult, 6–8 week-old, male Brown Norway rats were obtained from the National Laboratory Animal Center (Taipei, Taiwan). These animals were raised in normal conditions in the animal center of Changhua Christian Hospital. The study was approved by the Institutional Animal Care and Use Committee of Changhua Christian Hospital. Tracheas and bronchi were collected. Tissues were incubated in Hanks’ balanced salt solution (HBSS) with 0.1% collagenase solution (Sigma Chemical Co., St. Louis, MO, USA) at 37 °C for 20 min. Loosened connective tissues were scraped. The tissues were cut into 0.5-mm^3^ fragments, which were placed in a culture dish containing Dulbecco’s modified Eagle’s medium (DMEM)/F12, 10% fetal bovine serum (FBS), 2 mM glutamine, and 100 U/mL penicillin/streptomycin (all from Invitrogen, Carlsbad, CA, USA). They were then incubated at 37 °C. Passages six to eight were used for the experiments. Cultured ASMCs expressing smooth muscle actin were detectedby smooth muscle actin antibody immunocytochemically.

### 4.2. Evaluation of Cell Proliferation and Cell Cycle Progression

XTT labeling mixture reagents were used (cell proliferation Kit II, Roche Molecular Biochemicals, Indianapolis, IN, USA). Growth-arrested cells were stimulated with recombinant rat PDGF-BB (40 ng/mL; R&D Systems, Minneapolis, MN, USA) in the presence or absence of oligo-fucoidan (100–1000 μg/mL) (Hi-Q oligo-fucoidan^®^ as gift from Hi-QMarine Biotech International Ltd., Taipei, Taiwan) for 24 h, 48 h, or 72 h. XTT mixture reagent was added to each 96-well and incubated for 4 h; the absorbance at 490 nm was measured. Growth-arrested cells were stimulated with PDGF-BB in the presence or absence of oligo-fucoidan for 24 h to examine cell cycle progression. The harvested cell pellet was added to 3 mL of cold 70% ethanol and maintained at −20 °C for 30 min. The cell pellet was resuspended with 1% Triton X-100, 0.1 mg/mL RNase A, and 4 g/mL propidium iodide after centrifugation. Flow cytometry (FC 500, Beckman Coulter, Inc., Fullerton, CA, USA) was used to examine cell cycle progression.

### 4.3. Apoptosis Assay

Annexin V-FITC apoptosis detection kit (Strong Biotech Corporation, Taipei, Taiwan) was used to determine the percentage of necrotic cells. Briefly, the treated cells were washed with PBS and centrifuged at 200 g for 5 min. The cell pellet was resuspended in staining buffer for 15 min at 25 °C and were used for flow cytometric analysis. Annexin V-positive and PI-positive staining indicated apoptosis and necrosis, respectively.

### 4.4. Extraction of Protein and Western Blot Analysis

Total cellular proteins were extracted using a lysis buffer and cytoplasm and nuclear proteins were obtained using NE-PER reagents (Pierce Biotechnology, Rockford, IL, USA). The protein concentration was determined using Bio-Rad protein assay (Bio-Rad, Hercules, CA, USA). Antibodies against, Akt, Ser473-phosphorylated Akt, phosphorylated ERK1/2, ERK1/2, phosphorylated NF-κB, NF-κB, and α-actin were purchased from Cell Signaling Technology (Beverly, MA, USA). Secondary antibodies were purchased from Pierce Biotechnology. Cell proteins were fractionated by electrophoresis on a 10% SDS-polyacrylamide gel and were transferred onto a nitrocellulose membrane, blocked, and probed with various primary antibodies. Following incubation with primary antibodies (1:1000) overnight, the membrane was washed and incubated with horseradish peroxidase-conjugated secondary antibody (1:10,000) for 1 h. The blot was washed and visualized by enhanced chemiluminescence (Pierce Biotechnology). The bands were quantified and normalized against α-actin.

### 4.5. Statistical Analysis

Statistical analyses were carried out using SPSS/Windows software (SPSS Science, Chicago, IL, USA). The statistical significance was evaluated by one-way ANOVA and post hoc Tukey’s test, *p* < 0.05 indicate significant differences.

## 5. Conclusions

Oligo-fucoidan supplementation might reduce proliferation of PDGF-treated ASMCs by suppressing ERK1/2 and Akt phosphorylation and NF-κB activation, thereby upregulating the proliferation of ASMCs. The results of this study could be the basis of future animal experiments and human trials.

## Abbreviations

The following abbreviations are used in this manuscript

ASMCs: Airway smooth muscle cells
AHR: airway hyper-responsiveness
CAMs: cell adhesion molecules
DMEM: Dulbecco’s modified Eagle’s medium
ERK1/2: extracellular signal-regulated kinase
FBS: fetal bovine serum
GM-CSF: granulocyte monocyte-colony stimulating factor
HBSS: Hanks’ balanced salt solution
IL: interleukin
ICAM: intercellular CAM
ICS: inhaled corticosteroids
IgE: immunoglobulin E
LABA: long-acting beta2-agonists
LIF: leukemia inhibitory factor
MCP: monocyte chemoattractant protein
MAPK: mitogen activated protein kinase
(NF)-κB: nuclear factor
PDGF: Platelet-derived growth factor
PI3K: phosphoinositide-3-kinase
TNF: tumor necrosis factor
VCAM: vascular CAM
